# Jansen's Disease: Bone Abnormalities Beyond Chondrodysplasia

**DOI:** 10.1210/clinem/dgaf097

**Published:** 2025-02-14

**Authors:** Renata C Pereira, Anne M Delany, Monica Reyes, Barbara Gales, Harald Jüppner, Isidro B Salusky

**Affiliations:** Pediatric Nephrology, UCLA, Geffen School of Medicine, Los Angeles, CA 90095-8347, USA; Center for Molecular Oncology, UConn Health, Farmington, CT 06030, USA; Endocrine Unit, Massachusetts General Hospital and Harvard Medical School, Boston, MA 02114, USA; Pediatric Nephrology, UCLA, Geffen School of Medicine, Los Angeles, CA 90095-8347, USA; Endocrine Unit, Massachusetts General Hospital and Harvard Medical School, Boston, MA 02114, USA; Pediatric Nephrology Unit, Massachusetts General Hospital and Harvard Medical School, Boston, MA 02114, USA; Pediatric Nephrology, UCLA, Geffen School of Medicine, Los Angeles, CA 90095-8347, USA

**Keywords:** Jansen's disease, bone biopsy, immunohistochemistry, FGF23, osteocyte

## Abstract

**Context:**

Jansen metaphyseal chondrodysplasia (JMC) is an ultra-rare autosomal dominant disease that is caused by heterozygous, activating PTH1R mutations resulting in PTH- and PTHrP-independent hypercalcemia and hypercalciuria, leading to nephrocalcinosis and impaired renal function later in life. The activated PTH1R plays critical roles in mineral ion homeostasis and bone lengthening, as well as bone formation and resorption. Currently, little is known about bone turnover markers and bone histomorphometric changes in JMC patients.

**Objective:**

This study aimed to assess changes in bone microarchitecture, bone formation, and bone protein expression in 2 pediatric patients with JMC harboring the H223R-PTHR1 mutation.

**Methods:**

Bone histomorphometry, immunohistochemistry, and histologic analyses were conducted on iliac crest biopsy samples from 2 male siblings affected by JMC (ages 6 and 8 years) and 9 healthy control males of similar age, with normal kidney function.

**Results:**

Both patients with JMC displayed irregular bone architecture, increased osteoid, and a prolonged osteoid maturation process. While trabecular volume remained normal, immunohistochemical analysis demonstrated increased in PTH1R expression in both osteoblasts and fibroblastic cells on the bone surface. Cortical bone displayed areas of intense osteoclast activity and scattered marrow fibrosis. Remarkably, osteocytes in samples from patients with JMC had osteoid buildup within their lacunae and canaliculi that were both shorter and less abundant. DMP1 immunohistochemistry highlighted the abnormal canalicular network in patients. FGF23 staining in osteocytes was enhanced while sclerostin was diminished.

**Conclusion:**

The H223R-PTH1R mutation in patients with JMC leads to bone structural irregularities, hypomineralization, abnormal osteocyte morphology, and altered expression of osteocyte-derived proteins. These findings underscore the multifaceted impact of the mutant PTH1R on bone physiology and focus attention on the osteocyte as a cellular target for therapeutic intervention. Whether normalizing gene expression in osteocytes is possible and can improve bone health in patients with JMC remains to be seen. Assessment of osteocyte morphology and function may provide novel diagnostic endpoints for future clinical trials with JMC therapeutics.

The PTH/PTHrP receptor (PTH1R) mediates the endocrine actions of parathyroid hormone (PTH) and the paracrine actions of PTH-related peptide (PTHrP). It is abundantly expressed in kidney, osteoblasts, and growth plate chondrocytes. When this G protein–coupled receptor is activated in the proximal renal tubules, PTH increases urinary phosphate excretion by reducing expression of the sodium-dependent phosphate transporters type 2a and 2c. By enhancing 1-α-hydroxylase activity in this section of the kidney, PTH also increases the formation of 1,25(OH)_2_ vitamin D, thereby increasing intestinal calcium absorption. In distal renal tubules, the activated PTH1R enhances calcium reabsorption (1). PTH1R is also expressed in prehypertrophic growth plate chondrocytes, slowing their differentiation into hypertrophic cells through the paracrine actions of PTHrP, thereby contributing to the regulation of linear bone growth (2, 3). In osteoblasts, activation of the PTH1R by PTH directly affects osteoblast and osteocyte activity, and it indirectly enhances, through the RANK/RANKL system, osteoclast maturation and activity. Thus, the activated PTH1R plays critical roles in mineral ion homeostasis and bone lengthening, as well as bone formation and resorption.

Jansen's metaphyseal chondrodysplasia (JMC) is an ultra-rare autosomal dominant disease that is caused by heterozygous, activating PTH1R mutations (4-6) resulting in PTH- and PTHrP-independent hypercalcemia and hypercalciuria, leading to nephrocalcinosis and impaired renal function later in life (6, 7). Currently, 5 different PTH1R mutations, affecting 1 of 3 different amino acid residues, are known to cause JMC; these mutations, H223R, T410P/R, and I458 K/R, are located at the intracellular end of transmembrane helices 2, 6, and 7, respectively, and cause agonist-independent cAMP formation (6). In the growth plate, constitutively active PTHR1 mutations slow chondrocyte maturation, leading to marked abnormalities that resemble severe rachitic changes (8-10). In addition to short stature and bowing of long bones, patients with JMC often exhibit micrognathia, hypertelorism, high-arched palate, scoliosis, delayed tooth eruption or impaction, and premature closure of cranial sutures (6).

Currently, little is known about bone turnover markers and bone histomorphometric changes in patients with JMC. In fact, bone turnover, as well as cortical and trabecular bone parameters, and the extent of unmineralized osteoid have been assessed thus far only in a single adult patient with the T410P mutation (7). The purpose of the current study was to assess bone histomorphometry as well osteocyte abnormalities in 2 pediatric patients, brothers with the H223R mutation, and to compare these findings to healthy controls of similar age and with normal kidney function.

## Materials and Methods

### Patients

The bone features of the 2 brothers with the H223R mutation (JMC1 and JMC2) were investigated at the ages of 6 and 8 years at UCLA. Some of the clinical and laboratory information had been previously reported (11). In summary, the older brother at 2 years and 4 months and at 6 years of age displayed mild hypercalcemia, with normal serum phosphate levels, and undetectable or reduced PTH levels. At both ages, total alkaline phosphatase activity was elevated considerably, as was the urinary calcium to creatinine ratio. His younger brother had a normal serum calcium level when first tested at the age of 40 days, whereas serum phosphate was age appropriate and PTH was at the lower end of the normal range. By 4 years of age, he had become mildly hypercalcemic, phosphate remained within normal limits, and PTH was then well below the normal range. Total alkaline phosphatase activity was elevated at both ages, as well as the urinary calcium to creatinine ratio. We intended to assess bone histology and turnover parameters in treatment-naïve JMC; both patients had therefore not been treated with a bisphosphonate or vitamin D supplements.

### Tetracycline-Labeled Iliac Crest Bone Biopsies

Patients with JMC and controls were administered tetracycline at 10 mg/kg per day for 2 consecutive days. Following a 14-day interval, they were given tetracycline again at 10 mg/kg per day, for 2 days, as previously described (12). Bone biopsy was performed 4 days after the last tetracycline dose. The control population consisted of healthy children with normal kidney function who were undergoing minor elective orthopedic or urological surgery. Histomorphometric parameters for the controls were previously reported (13). From this cohort we selected 9 subjects within the age range of 4.5 to 12 years.

Full thickness bone biopsies were obtained from the anterior iliac crest (2 cm below the anterior superior iliac spine) using a modified Bordier trephine needle under general anesthesia. Biopsy specimens were 0.5 cm in diameter by 0.5 to 0.6 cm in length. Specimens were dehydrated in alcohol, cleared with xylene, and embedded in methylmethacrylate. Static histomorphometric parameters were evaluated in undecalcified 5-μm sections stained with Toluidine blue; dynamic histomorphometric parameters were assessed in unstained 10-μm sections (12). Additional trichrome blue staining was performed to better visualize osteoid accumulation using the Trichrome Stain Kit (Abcam) according to manufacturer's instructions.

Primary bone histomorphometric parameters were assessed in trabecular bone under 200× magnification using the OsteoMeasure software (OsteoMetrics, Decatur, GA). Mineralized bone was defined by purple/blue-staining areas; pale, blue-stained seams at least 1.5 μm in width were included in measurements of unmineralized bone (osteoid). Polarized light microscopy was used to visualize cortical osteons. Cortical thickness (Ct.Th µm) was also quantified using the OsteoMeasure; values from both cortices were obtained and data from both patients were pooled. All variables were calculated according to American Society for Bone and Mineral Research recommendations (14). The reported normal values for cortical thickness were obtained from data published by (15), while normal values for osteoblast and osteoclast surface were obtained from Glorieux et al (16).

### Biochemical Parameters

Biochemical determinations for calcium (Ca), phosphorus (P), alkaline phosphatase, intact PTH, 25(OH) vitamin D, 1,25(OH)_2_ vitamin D, and urinary N-telopeptide cross-links were measured by PhysLab (Omaha, NE). Serum intact and C-terminal FGF23 levels were determined in plasma using human “intact” and “C-terminal” immunoassays (Immutopics International, San Clemente, CA). The intact assay is a 2-site enzyme-linked immunosorbent assay using antibodies directed against the carboxyl- and amino-terminal portions of the molecule, measuring exclusively full-length FGF23 in plasma. The C-terminal enzyme-linked immunosorbent assay uses 2 antibodies directed against the carboxyl-terminal portion of the molecule and detects both total intact (RRID:AB_2891250) and C-terminal FGF23 (RRID:AB_2722648). The reported normal ranges for intact and C-terminal FGF23 in healthy children were obtained from our own cohort (17).

### Detection of Protein Expression in Bone

The technique for immunohistochemical detection of protein in bone was adapted from a previously reported method (18). In brief, 5-μm sections of bone tissue were deplasticized in xylene and chloroform, rehydrated in graded alcohol solutions, and partially decalcified in 1% acetic acid. Endogenous peroxidase activity was quenched in 3% hydrogen peroxide/methanol solution. Nonspecific binding was blocked in avidin–biotin solution and in 5% normal horse serum with 1% bovine serum albumin. Sections were incubated with affinity purified polyclonal goat antihuman FGF23 (225-244) (Immutopics International) (dilution 1:500), monoclonal antihuman DMP1 (RRID:AB_2292808) (LFMb31) (62-513) (Santa Cruz Biotechnology, Inc., Santa Cruz, CA) (dilution 1:50), monoclonal antihuman sclerostin (RRID:AB_2195350) (R&D Systems, Minneapolis, MN) (dilution 1:500), rabbit anti-PTH1R (RRID:AB_3675574) (dilution 1:200) (kindly provided by Dr. Peter Friedman, University of Pittsburgh School of Medicine) (19). Sections were incubated with primary antibody overnight at 4 °C in a humidified chamber. Sections were then incubated with biotinylated antigoat (RRID:AB_2336123) (Vector Laboratories, Burlingame, CA), antimouse (RRID:AB_2313581) (Sigma-Aldrich, St. Louis, MO), or antirabbit (RRID:AB_2313606) (Sigma-Aldrich) antibody. To visualize antigens, slides were incubated for 30 minutes with StreptABC Complex/HRP kit (Vector Laboratories) followed by AEC substrate–chromogen (Dako, Carpinteria, CA); and counterstained with Mayer's hematoxylin (Sigma-Aldrich). Immunofluorescence was performed to colocalize expression of FGF23 and DMP1 using the goat antihuman FGF23 (RRID:AB_3675567) (225-244) and mouse antihuman DMP1 primary antibodies, respectively, and Alexa Fluor conjugated antimouse Alexa 488 (RRID:AB_2536087), and antigoat-Alexa 594 (RRID:AB_2534105) (Invitrogen, Carlsbad, CA) secondary antibodies. DAPI staining was used to assess numbers of viable osteocytes in trabecular bone. Negative controls were performed for each bone section by omitting the primary antibody. Sections were batched; thus, immunohistochemistry was performed simultaneously on the patient specimens, along with sections from healthy controls as well as negative controls, as previously reported (19). Reproducibility was ensured by repeating the immunohistochemical analysis on all specimens. To quantify the bone expression of FGF23, whose presence in the boney trabeculae were limited to the osteocyte cell bodies, staining was dichotomized as either “present” or “absent” in any given osteocyte, and the total number of trabecular osteocytes with positive staining was counted and normalized by tissue (T) or bone area (B.Ar).

### Ploton Silver Staining to Visualize Osteocyte Morphology

To visualize the osteocyte morphology and canalicular networks, plastic embedded bone sections were de-plasticized and rehydrated, then Plonton silver staining and cresyl violet counterstaining was performed as described by Dole et al (20). The CellSens program was utilized to quantify both the length and number of osteocyte canaliculi. Quantification of canaliculi number and length was conducted on all osteocytes where the nuclei were observed. The data are presented as intervals for length or number, and the distribution within each interval is expressed as a percentage of the total number of osteocytes quantified. The data presented are averaged for each patient. For both patients with JMC, 233 and 134 osteocytes, respectively, were quantified, for a total of 980 canaliculi measured. For normal controls, data from 4 individuals were pooled. For each control sample, between 72 and 139 osteocytes were quantified, with a total of 3578 canaliculi measured. The Fisher exact test was used to determine whether the distribution of canaliculi length or number was significantly different between controls and patients.

### Osteocyte Phenotype in Humanized PTHR1 H223R Mouse Model

To generate “humanized” PTHR1 mice, the exons encoding the membrane-embedded portion, the intracellular C-terminal tail, and most of the N-terminal extracellular domain of the murine *Pthr1* gene were replaced with the corresponding sequences of the human *PTHR1* cDNA, so that the human PTHR1 will be expressed under the control of the endogenous *Pth1r* promoter (21). After mating females that were homozygous for the “humanized” PTH1R with wild-type males, the H223R mutation was introduced into the “humanized” PTH1R allele of fertilized eggs to generate JMC mice that are heterozygous for H223R-PTH1R mutant (22). Femur specimens were obtained from male H223R-PTH1R mice at 42 days of age, which underwent a series of preparation steps similar to those performed on the human bone samples prior to embedding in methylmethacrylate. Sections (5-μm) were obtained and subjected to Ploton silver staining as described above.

## Results

### Biochemical Parameters

The 2 siblings, JMC1 (6 years old) and JMC2 (8 years old), displayed serum phosphate levels within the age-appropriate normal range, while total serum calcium levels were at the upper end of the normal range. The patients with JMC had higher urinary calcium excretion, likely due to higher filtered load of calcium. PTH levels were undetectable while alkaline phosphatase levels were prominently elevated in both patients. Mild increases in intact and C-terminal FGF23 as well as 1,25(OH)_2_ vitamin D were noted (Table 1). These laboratory findings are consistent with those previously described for other patients with JMC (6, 7) and for JMC1 and JMC2 at a younger age (11).

**Table 1.** Serum biochemistry in patients with JMC compared with healthy controls

	JMC1 (6 years old)	JMC2 (8 years old)	Normal ranges in children
C-FGF23 (RU/mL)	100	98	49 ± 13
Intact FGF23 (pg/mL)	56	62	33 (12.7,98.1)
Phosphorus (mg/dL)	3.2	3.5	2.3-5.7
Calcium (mg/dL)	10.6	10.5	8.5-10.5
1,25-D (pg/mL)	82.3	70	20-79
25-D (ng/mL)	24	18	30-100
Intact PTH (pg/mL)	< 2.5	< 2.5	14-72
Alkaline phosphatase (IU/L)	632	564	<370
Urine NTx (nM Bce/mM creatinine)	1212	1757	167-578

Reference data for C-FGF23 (mean ± SD) and intact FGF23 (median and range) in normal healthy children were obtained from our own cohort (17).

Abbreviations: JMC, Jansen metaphyseal chondrodysplasia; NTx, N-telopeptide cross-link; PTH, parathyroid hormone.

### Bone Histology and Histomophometric Analysis

**Figure 1.**
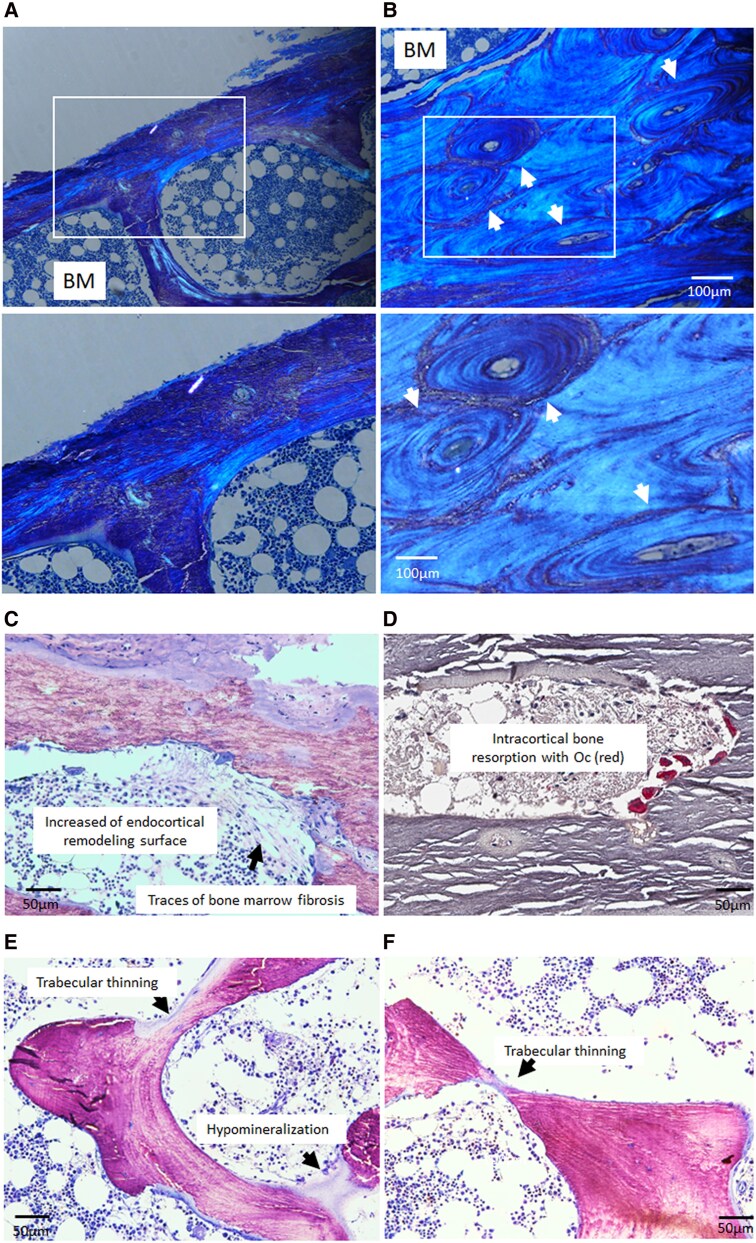
Bone histological alterations in patients with JMC compared with age-matched controls with normal kidney function. Both brothers showed similar bone histology features and representative images are shown. Sections of iliac crest biopsies were analyzed by polarized light microscopy, demonstrating cortical thinning and decreased osteons in (A) JMC patient compared with (B) control. The lower panels are higher magnification images of upper panels. Osteons in controls are indicated by white arrows. BM, bone marrow. (C) Increased remodeling of the endocortical surface, with regions of bone marrow fibrosis (arrow) in patients with JMC. (D) Increased intracortical resorption with active osteoclasts (Oc) stained red for TRAP in patients with JMC. (E, F) Toluidine blue stained trabecular bone from patients with JMC. Mineralized bone stains purple and osteoid (hypomineralized bone) stains pale blue. Areas of hypomineralized bone and trabecular thinning are indicated by the arrows.

Histological examination of bone samples from JMC1 and JMC2 revealed marked alterations in the microarchitecture of cortical bone. Specifically, cortical bone from patients with JMC was considerably thinner. The average cortical thickness from both patients with JMC was 180 ± 17 µm compared with 651 ± 119 µm in similarly aged normal controls (15). Moreover, the cortical bone displayed a depletion of osteons, the concentric layers of compact bone that comprise the functional units of cortical bone (Fig. 1A and 1B). We also observed increased osteoclast activity on endocortical and intracortical surfaces resulting in cortical perforations. Moreover, scattered areas of bone marrow fibrosis were observed on the endocortical surfaces, which were not seen in normal controls (Fig. 1C and 1D). Additionally, hypomineralization (Fig. 1D) and areas of thinning (Fig. 1E) were observed in the trabecular bone of patients with JMC, which could lead to trabecular perforations. Altogether, hypomineralization, decreased cortical thickness, altered microarchitecture and increased remodeling activity in patients with JMC would be consistent with increased bone fragility (Fig. 1).

Dynamic bone histomorphometry analysis (Table 2) demonstrated that although bone formation rate (remained within the normal range, the patients with JMC had decreased adjusted apposition rate, an indication of impaired osteoblast activity. This was accompanied by an increase mineralization lag time and osteoid maturation time, indicating a delay in the mineralization processes. Together, these data suggest subtle disruptions in bone quality and remodeling dynamics that are characteristic of hypomineralization, which may reflect an early-stage of metabolic bone disorder.

**Table 2.** Structural, static and dynamic bone histomorphometric characteristics in iliac crest biopsy from patients with JMC compared with those in healthy male controls (mean ± SD)

	JMC1	JMC2	Healthy controls (N = 9)
Age	6 years	8 years	4-12 years
Bone volume (BV/TV) (%)	24.9	17.9	16.1 ± 4.32
Osteoid volume (OV/BV) (%)	5.7	4.0	1.4 ± 0.88
Osteoid thickness (μm)	8.1	7.7	6.3 ± 4.1
Osteoid surface (OS/BS) (%)	31.7	23.3	10.4 ± 4.42
Osteoblast surface (Ob.S/BS) (%)	6	5.6	8.2 ± 4.4*^a^*
Eroded surface (ES/BS) (%)	5.3	3.7	4.1 ± 0.36
Osteoclast surface (Oc.S/BS) (%)	1.3	0.9	0.94 ± 0.38*^a^*
Trabecular thickness (μm)	88.4	92.1	81.2 ± 14.02
Trabecular separation (μm)	267.1	422.6	448.2 ± 134
Trabecular number (/mm)	2.8	1.9	2 ± 0.47
Mineralizing surface (MS/BS) (%)	3.7	4.8	4.65 ± 2.7
Mineral apposition rate (μm/day)	0.8	0.94	1.03 ± 0.27
Bone formation rate (BFR/BS) (μ^3^m/μ^2^m/y)	10.7	16.7	18.5 ± 12
Adjust apposition rate (Aj.AR) (μm/day)	0.09	0.19	0.43 ± 0.2
Mineralization lag time (MLT) (d)	87.7	39.1	12.7 ± 5.32
Osteoid maturation time (OMT) (d)	10.2	8.1	4.93 ± 1.3

Abbreviation: JMC, Jansen's metaphyseal chondrodysplasia.

^*a*^The normal values for Ob.S/BS and OcS/BS were obtained from Glorieux et al (age 7.0-10.9 yr) (16).

**Figure 2.**
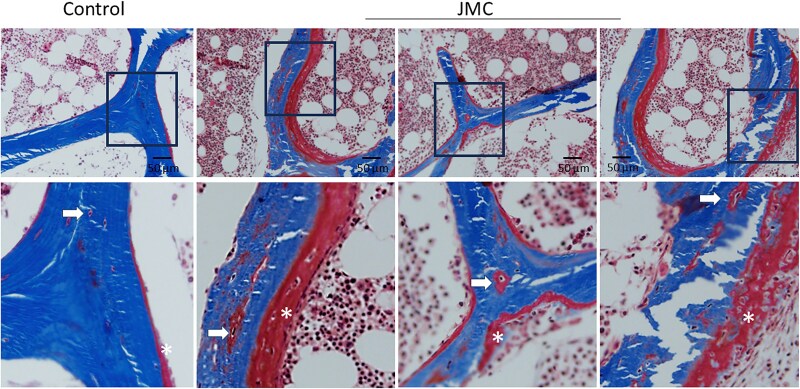
Abundant osteoid accumulation on trabecular surfaces (*) and osteoid halos surround osteocytes in patients with JMC (arrows). Representative images of Trichrome staining of iliac crest biopsies from a normal control (leftmost panel) and patients with JMC (remaining 3 panels). The lower panels are higher magnification images of the selected region in the upper panel. Mineralized bone stains blue while osteoid stains red.

Within cancellous bone, histomorphometry showed trabecular bone volume that remained within expected normal range (Table 2). However, both patients showed a pronounced increase in osteoid (unmineralized bone) volume and surface (Fig 2 and Table 2). This is congruent with the increase in osteoid maturation time and mineralization lag time, and the decreased adjusted apposition rate, thus reinforcing the concept that bone from the patients with JMC displays hypomineralization, and consistent with the observed elevated serum alkaline phosphatase levels. In addition to the broad areas of unmineralized bone on the trabecular surfaces, there were osteoid halos enveloping osteocytes, indicating that hypomineralization extends to osteocyte lacunae as well (Fig. 2).

### Osteocyte Morphology

**Figure 3.**
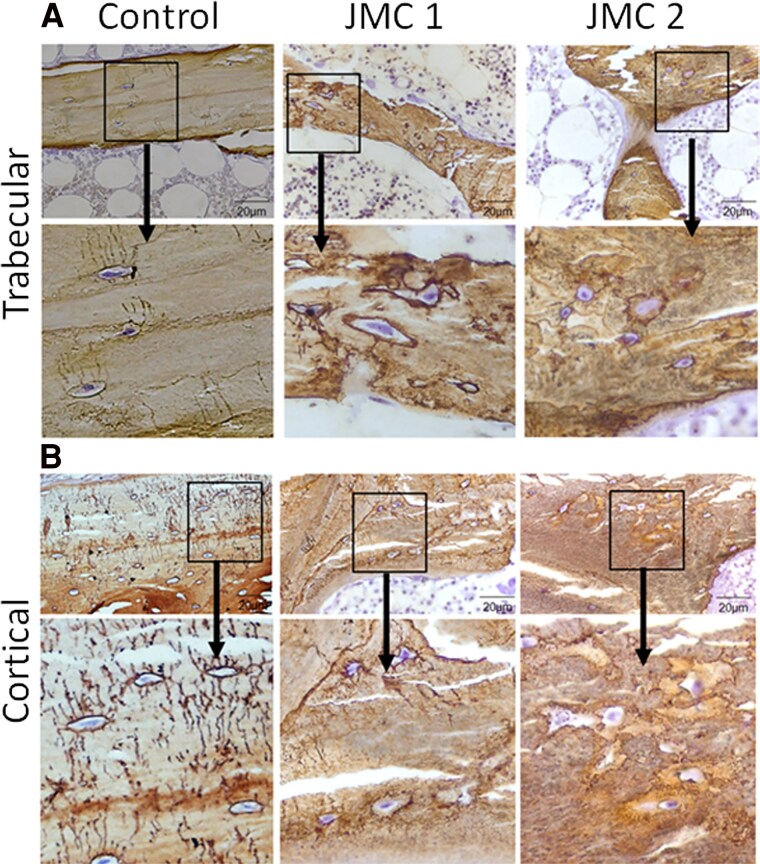
Ploton silver staining of nondecalcified iliac crest biopsies showing a severe defect in canalicular morphology of osteocytes in patients with JMC. Representative images of (A) trabecular bone and (B) cortical bone. The leftmost panels represent a normal control sample, while the center and rightmost panels are from patients with JMC. The lower panels are higher magnification images of the selected region in the upper panel. Nuclei are stained purple. In the control tissue, a normal network of osteocyte canaliculi and lacunae is observed, while the JMC samples show a marked reduction in the number and length of canaliculi.

**Figure 4.**
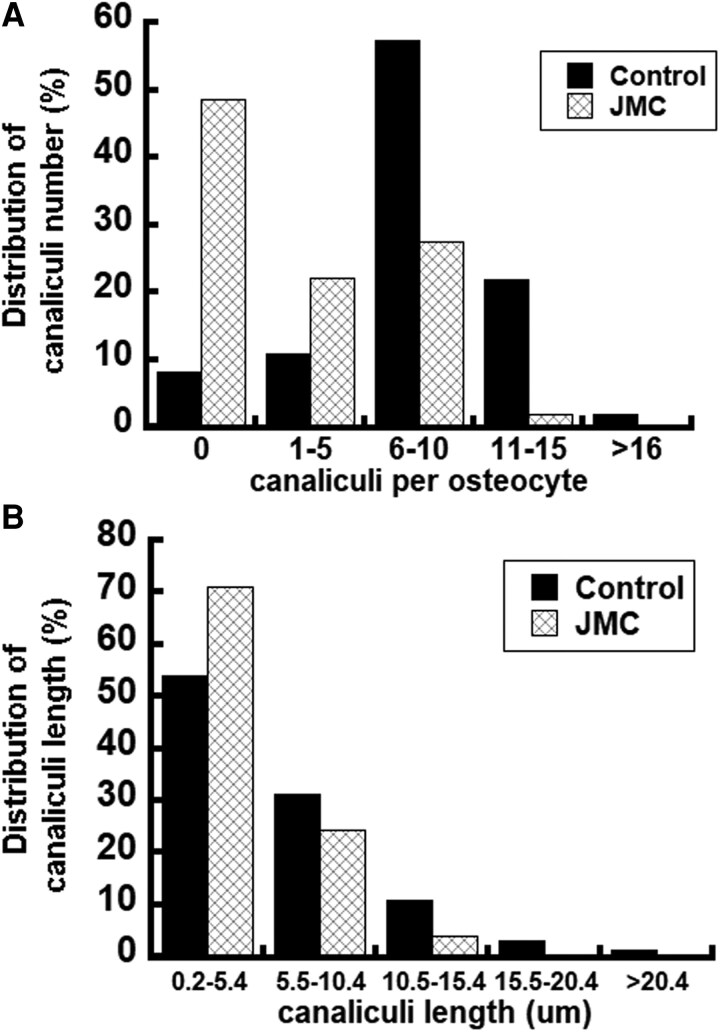
Analysis of trabecular canaliculi in patients with JMC and controls. Ploton silver-stained iliac crest biopsies from normal controls and patients with JMC were quantified for distribution of (A) osteocyte canaliculi numbers and (B) length. The data are presented as intervals for length or number, and the distribution within each interval is expressed as a percentage of the total number of osteocytes quantified in each interval. Pooled measurements from both patients and from 4 controls. Distribution of canaliculi number was significantly different between patients and controls (*P* = .00002), as was the distribution of canaliculi length (*P* = .0208).

Due to the observed hypomineralization within osteocyte lacunae, we further investigated whether potential abnormalities are present in osteocytes. While patients with JMC had more osteocyte lacunae than controls (Number of osteocyte/B.Ar 231 ± 31 for patients with JMC vs 146 ± 0.6 for controls), their canalicular networks housing these dendrites were also notably reduced within both cortical and cancellous bone (Fig. 3A and 3B). The trabecular canalicular length and number were quantified, which showed, remarkably, that nearly 50% of osteocytes in both patients displayed a failure to develop any dendritic projections (Fig. 4A). Additionally, approximately 70% of the canaliculi in patients with JMC had a length of less than 5.5 µm, while only approximately half of the canaliculi in control bone samples were that short (Fig. 4B). Defects in osteocyte canalicular number and length, coupled with the abundance of osteoid surrounding osteocyte bodies suggest that osteocyte function and lacunar remodeling are compromised in patients with JMC. Defects in osteocyte numbers and connectivity are seen in aging as well as diseases such as osteoporosis and osteogenesis imperfecta (23, 24).

### Bone Protein Expression Assessed by Immunostaining

**Figure 5.**
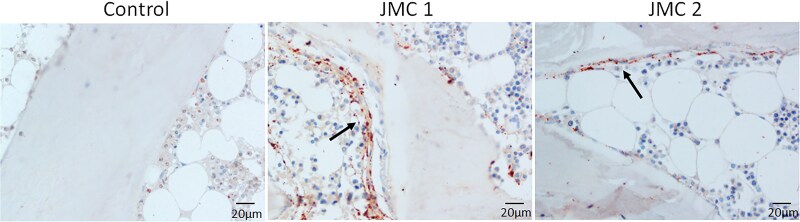
Expression of PTH1R in trabecular bone from control and patients with JMC. Representative images of iliac crest biopsies from a normal kidney function control (leftmost panel) and patients with JMC (center and right panels) subjected to immunohistochemical staining for PTH1R. Positive PTH1R staining appears red/brown, while nuclei are stained blue. Arrows point to abundant PTH1R-positive osteoblasts and fibroblastic cells on the bone surface in patients with JMC.

**Figure 6.**
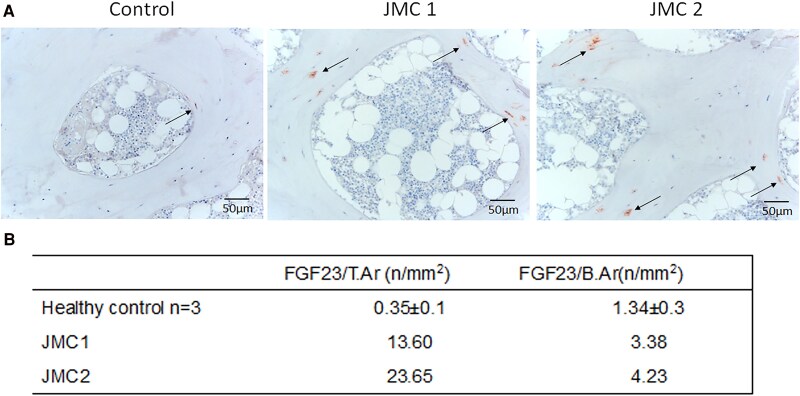
Immunohistochemical staining for osteocytes expressing FGF23 in cancellous bone. (A) Representative images of iliac crest biopsies from a normal kidney function control (leftmost panel) and patients with JMC (center and right panels) subjected to immunohistochemical staining for FGF23. Positive FGF23 staining appears red/brown, while nuclei are stained blue. Arrows highlight osteocytes exhibiting strong FGF23-positive staining. Note that FGF23-positive osteocytes are found primarily towards the surface of the trabeculae in the controls, but throughout the trabeculae in patients with JMC. (B) Quantified data for FGF23 staining, demonstrating a marked increase in FGF23-positive osteocytes in patients with JMC.

**Figure 7.**
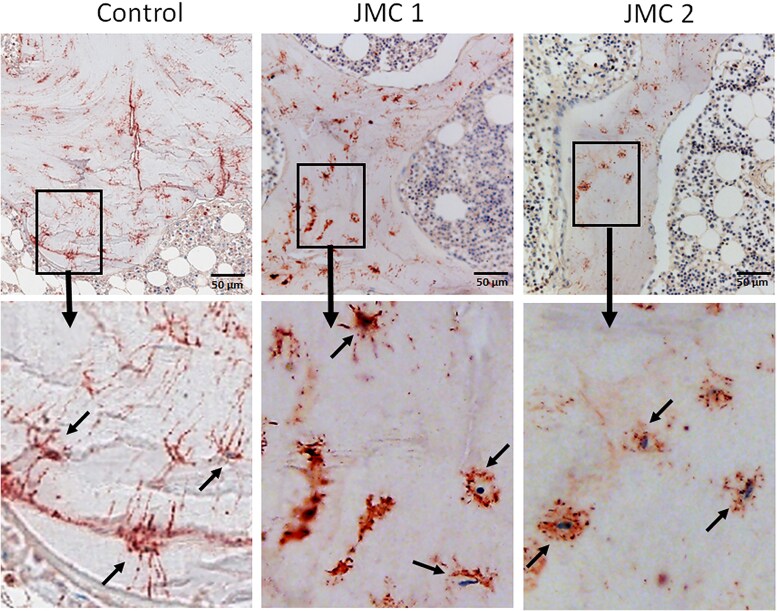
Immunohistochemistry for osteocytes expressing DMP1 in cancellous bone. Representative images of iliac crest biopsies from a normal kidney function control (leftmost panel) and patients with JMC (center and right panels) subjected to immunohistochemical staining for DMP1. Positive DMP1 staining appears red/brown, while nuclei are stained blue. The lower panels are higher magnification images of the selected region in the upper panel. Arrows highlight osteocytes exhibiting strong DMP1-positive staining. Note DMP1 staining of osteocyte lacunae and canaliculi in the controls, while DMP1 accumulated in the perilacunar region of the osteocyte in patients with JMC.

Immunohistochemistry of trabecular bone from both patients showed intense PTH1R staining in cells lining the bone surface, some osteocytes and layers of fibroblastic cells adjacent to the bone surface. In contrast, PTH1R staining seemed limited primarily to osteoblasts on the bone surface in controls (Fig. 5). FGF23 production by osteocytes, important for maintaining phosphate homeostasis, is typically elevated in patients with kidney disease (25-27). As expected, within the trabecular bone from patients with JMC, many more osteocytes stained positive for FGF23 protein than control samples (Fig. 6). DMP1 was abundantly expressed in osteocytes of control samples, with DMP1 protein evident within the lacunae and canaliculi (Fig. 7). In contrast, DMP1 staining in JMC osteocytes showed an accumulation of DMP1 in the perilacunar region and diffuse staining in canaliculi, strengthening the idea that osteocyte morphology and their likely function are affected in JMC.

**Figure 8.**
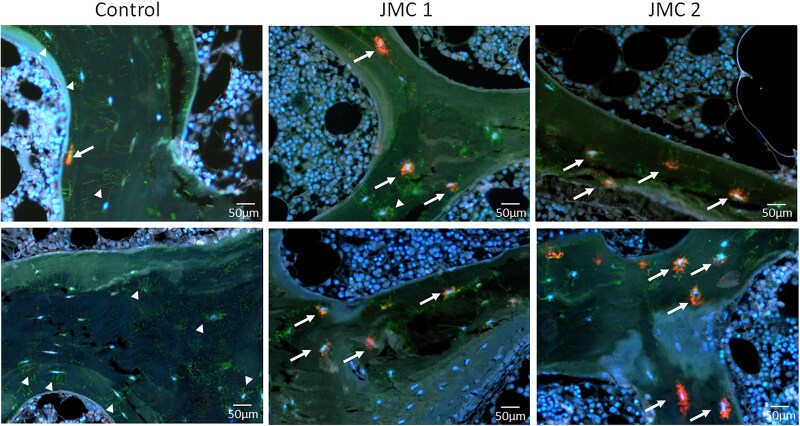
FGF23 and DMP1 are not colocalized in trabecular bone osteocytes. Two representative images of iliac crest biopsies from a normal kidney function control (leftmost panel) and patients with JMC (center and right panels) subjected to immunofluorescence staining. FGF23 signal is red, DMP1 signal is green, and nuclei are counterstained with DAPI (blue) to highlight live cells. Arrow heads indicate green DMP1-positive cells, while arrows indicate red FGF23-positive cells. Note the higher abundance of FGF23-positive cells in the patients with JMC.

**Figure 9.**
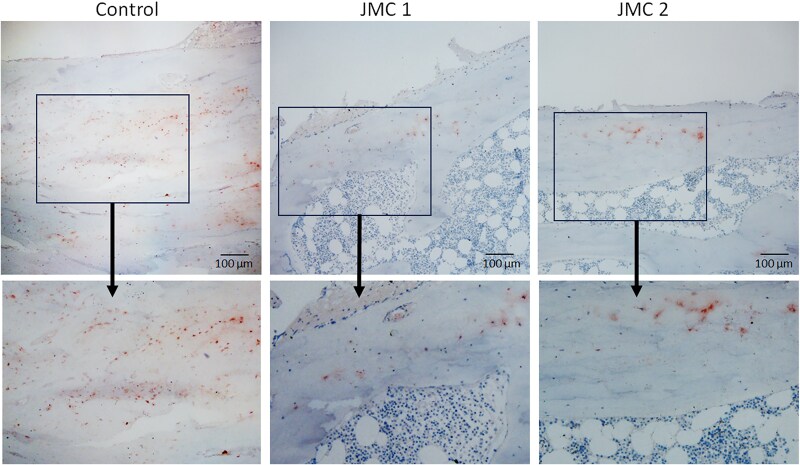
Immunohistochemistry for wnt signaling inhibitor sclerostin/SOST in cortical bone osteocytes. Representative images of iliac crest biopsies from a normal kidney function control (leftmost panel) and patients with JMC (center and right panels) subjected to immunohistochemistry for sclerostin. Positive sclerostin staining appears red/brown, while nuclei are stained blue. The lower panels are higher magnification images of the selected region in the upper panel. Sclerostin appears more abundantly expressed in osteocytes from controls compared to patients with JMC.

To determine whether DMP1 and FGF23 are expressed in the same osteocytes, dual immunofluorescence was performed (Fig. 8). In control samples, FGF23-positive osteocytes were sparse and those most strongly expressing FGF23 were primarily closer to the bone surface. In contrast, strongly staining FGF23-positive osteocytes were abundant in patients with JMC, which were found both near the bone surface and deeper within the trabeculae (Figs. 6 and 8). In controls, DMP1 staining was evident in canaliculi throughout the bone and within osteocyte lacunae, but this canalicular staining was much less pronounced in samples from patients with JMC (Figs. 7 and 8). There were few, if any, osteocytes that stained strongly for both DMP1 and FGF23 in either control or JMC samples (Fig. 8). This observation supports the concept that DMP1 is a local suppressor of FGF23 (28, 29). Lastly, we examined expression of sclerostin, a soluble Wnt inhibitor synthesized by mature osteocytes. Whereas sclerostin was not detected in cancellous bone, it was well expressed in cortical bone from controls, but there appeared to be fewer sclerostin-positive osteocytes in cortical bone from either patient (Fig. 9).

### Osteocyte Morphology in Humanized H223R-PTH1R Mice

Our findings on Jansen patient bone samples indicated abnormalities in osteocyte morphology. To corroborate these observations on the 2 affected brothers, we examined the osteocyte phenotype of a recently reported JMC mouse model in which the human PTH1R carrying the same H223R mutation as JMC1 and JMC2 is expressed under the control of the endogenous mouse *Pth1r* promoter (22). This knock-in mouse model reproduces key characteristics of patients with JMC, such as grossly enlarged growth plates, reduced long bone length, and reduced serum PTH and P1NP levels. One particularly striking feature is the delayed maturation of growth plate chondrocytes, evidenced by an enlarged proliferative zone and a diminished layer of hypertrophic chondrocytes. There are only very limited histological analyses of growth plate biopsies from patients with JMC, but the findings in H223R-PTH1R mouse model are consistent with the radiographic abnormalities typically observed in affected patients. Overall, these findings mirror the skeletal abnormalities typical of JMC (11). Further, JMC H223R mice had normal serum calcium and phosphate levels but suppressed PTH (1-84), as seen in JMC1 and JMC2. However, unlike our patients with JMC, femurs from H223R mutant mice had reduced cancellous bone, and intact but mildly reduced cortical bone. One other shortcoming of the model is the fact that even heterozygotes display a very short lifespan due to respiratory compromise (22).

**Figure 10.**
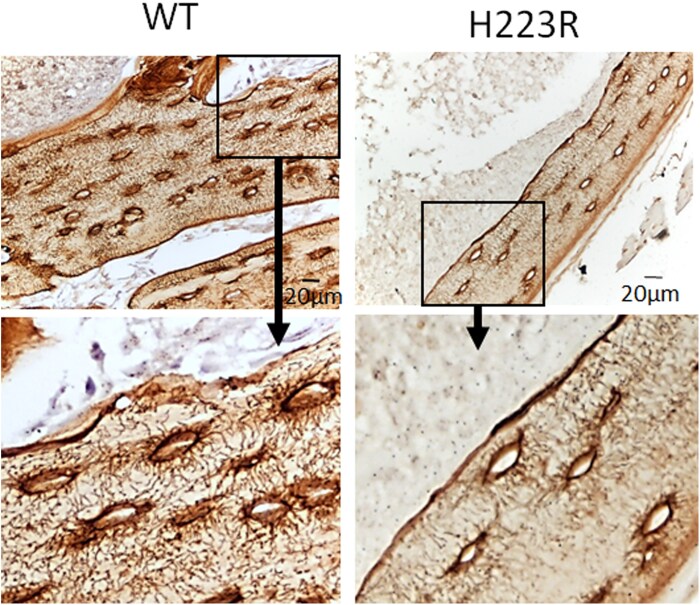
Osteocyte canalicular morphology in cortical bone of PTH1R H223R mice mimics that of patients with JMC. PTH1R H223R mice knock-in mice express a human PTH1R allele carrying the same H223R mutation as JMC1 and JMC2, expressed under the control of the endogenous mouse *Pth1r* promoter. Representative silver-stained sections of a non-decalcified femur from wild type (WT) control and H223R knock-in mice demonstrates a dramatic reduction in the number of osteocyte canaliculi in H223R compared to wild-type controls. The lower panels are higher magnification images of the selected region in the upper panel.

Nonetheless, the H223R-PTH1R mice offer a valuable framework for studying how constitutively active PTH1R signaling affects osteocytes. Ploton silver staining was performed, with analysis focusing on cortical bone, which demonstrated that osteocyte canalicular abnormalities in the H223R-PTH1R mice were similar to those observed in the JMC bone samples, while osteocytes in control mice were phenotypically normal (Fig. 10). These findings provide another level of validation for the H223R-PTH1R mouse model as a tool for studying JMC. In fact, this could open pathways for testing therapeutic interventions targeting osteocyte function while avoiding the ethical and practical challenges of obtaining sufficient bone samples from Jansen patients, especially given the rarity of the disease.

## Discussion

With the goal of further characterizing the skeletal lesions and the cellular phenotype that may underly their bone pathology, we report bone histomorphometric analyses of 2 pediatric patients with JMC with the H223R-PTH1R mutation. These patients displayed cortical bone thinning and increased osteoclast activity on the endocortical surfaces and severe hypomineralization in cancellous bone. In addition, a striking defect in osteocyte morphology was observed, including an accumulation of osteoid within the lacunae and dramatically reduced canalicular length and number. The osteocyte phenotype found in our patients with JMC was recapitulated in a humanized mouse model carrying the same H223R-PTH1R mutation. These observations indicate that activating mutations in PTH1R impair not only maturation of growth plate chondrocytes (22) but also the activity of osteoblasts and osteoclasts, impacting the overall bone remodeling process. Furthermore, the H223R-PTH1R mutation has a profound impact on osteocyte biology, likely contributing to the complex pathophysiology seen in patients with JMC.

Circulating intact PTH levels were undetectable in the patients with JMC due to a sustained increase in serum calcium levels caused by the activating PTH1R mutation. Excess PTH1R signaling enhances osteoblast and osteoclast activity, resulting in increased serum alkaline phosphatase and urine N-telopeptide cross-links, respectively (Table 1). However, new matrix formation was disordered, resulting in excess osteoid and regions of marrow fibrosis (Table 2 and Figs. 1C and 2) (30), which can weaken bone structure and contribute to bone deformities.

Cortical bone from patients with JMC displayed prominent osteoclast activity, although eroded surface in cancellous bone was not remarkably different from controls (Fig 1 and Table 2). Osteocytes are abundant in cortical bone, and they are a major source of RANKL, the cytokine needed for differentiation of bone-resorbing osteoclasts. The mechanisms underlying transcriptional activation of the RANKL gene downstream of PTH1R activation and cAMP production are well defined (31). Although RANKL levels were not quantified in our patients, the increased presence of osteoclasts on the endocortical surface reflects increased RANKL production mediated by PTH1R signaling. Whether there is a concomitant decrease in synthesis of the RANKL decoy osteoprotegerin has yet to be established.

Canonical Wnt signaling is important for bone anabolism, and the soluble Wnt antagonist sclerostin, encoded by the SOST gene, is a target of PTH1R signaling. The DNA elements and transcription factors responsible for downregulation of SOST transcription by PTH have been described (31) and the presence of fewer sclerostin-positive osteocytes in the patients with JMC is consistent with hyperactive PTH1R activity (Fig 9).

FGF23 is an osteocyte product playing a key role in mineral homeostasis, acting in the kidney to increase phosphate excretion (32). In the patients with JMC, serum phosphate levels were at the lower end of the normal range, while serum levels of both intact and C-terminal FGF23 were elevated (Table 1). This finding correlates with increased FGF23-positive osteocytes in the patients with JMC compared with controls (Fig 6).

Expressed by osteocytes, DMP1 limits FGF23 expression by mechanisms involving FGFR signaling and DMP1 is also important for nucleating mineralization (28, 33). While DMP1 is readily detected in JMC patient osteocytes, its distribution remained perilacunar, perhaps due to the paucity of canaliculi in the patients, suggesting a defect in its ability to be appropriately trafficked (Figs. 7 and 8). Indeed, the patients with JMC had a pronounced increase in unmineralized matrix in the metabolically active cancellous bone compartment and in the osteocyte lacunae themselves. This phenotype is similar to that observed in patients with homozygous inactivating DMP1 mutations, who have histological and immunohistochemical findings like those observed in our patients with JMC, including increased expression of FGF23 in focal bone areas, hypomineralization and increased osteoid volume (34). Thus, the altered distribution and trafficking of DMP1 likely contributes to JMC pathology. Moreover, whether DMP1 is appropriately cleaved to its active form in patients with JMC has not been investigated.

Osteocytes are the most abundant cells in mature bone tissue (27). The dendritic processes of osteocytes within their canaliculi form an extensive network throughout the bone matrix, allowing for communication and signaling between neighboring cells, bone surfaces, and blood vessels. Alterations in osteocyte networks and function such as those found in patients with JMC may disrupt the flow of nutrients and signaling molecules (ie, RANKL, sclerostin, FGF23, DMP1). In addition, osteocytes are the cells that sense mechanical strain, orchestrating bone remodeling and modeling processes in response to loading. The reduced number and length of dendritic processes in the osteocytes in patients with JMC and in the H223R-PTH1R mice (Figs. 4 and 10) suggests an altered ability of the osteocytes to sense and respond to mechanical loading, which could contribute to the bone-related complications observed in JMC.

In patients with JMC, the decreased osteocyte dendrite length and number may be related to increased ligand-independent cAMP signaling of the activated PTH1R mutant. In vitro studies utilizing osteocytic cell lines showed that PTH treatment decreased their dendritic morphology, which is similar to data obtained from rats injected with supraphysiological levels of PTH (35, 36). Our observations in patients and mice with the H223R-PTH1R mutation could be some of the first demonstrations of the PTH1R-mediated impact of osteocyte morphology in a physiological in vivo setting. Reduced dendrite number and connectivity is also seen in aged mouse bone, where this phenomenon precedes a decrease in osteocyte number (37). It is possible that patients with JMC may experience premature deficits in osteocyte number with aging.

The patients with JMC also displayed increased osteoid within the osteocyte lacunae (Fig. 2). Under physiologic conditions, osteocytic lacunar–canalicular remodeling occurs in response to PTH1R signaling during lactation via PTHrP and in exercise-induced remodeling via PTH (38). In this remodeling cycle, osteocytic osteolysis is facilitated by matrix-degrading proteases and by proton pumps acidifying the microenvironment; osteolysis is normally followed by replacement and mineralization of the matrix by the osteocyte. It is not clear whether the increased osteoid in lacunae of the patients with JMC is due to delays in mineralization or persistent local acidification. Nonetheless, the presence of osteoid in the osteocyte lacunae may impact bone mechanical properties (39, 40).

It remains uncertain whether increased bone resorption contributes to hypercalcemia. Treatment with bisphosphonates can ameliorate hypercalcemia by reducing osteoclast function, which may slow or prevent deterioration of kidney function. The subsequent addition of a thiazide has been reported to normalize blood calcium levels and to markedly reduce urine calcium excretion in patients with JMC (10, 41). Onuchic et al documented in 1 patient with the H223R-PTH1R mutation that the combination of alendronate (10 mg/day), initiated at 20 years of age, and hydrochlorothiazide initiated at 26 years of age (initially 12.5 mg/day, subsequently increased to 25 mg/day), normalized urinary calcium excretion. Although discontinuation of alendronate at the age of 31 years led to an increase in serum and urine calcium, despite treatment with a higher dose of hydrochlorothiazide (50 mg/day), the patient's renal function has thus far remained stable (10). While long-term outcome data for 5 additional patients with the H223R mutation who were treated with a bisphosphonate are not yet available, it appears plausible that limiting urinary calcium excretion could help preserve renal function.

Although JMC is a rare disease, its impact on patient quality of life and long-term health care burden emphasizes the need for an effective form of therapy. Presently, amino-terminally truncated PTH and PTHrP analogs with the Gly12→dTrp substitution, originally developed as PTH antagonists (42), hold some potential by functioning as inverse agonists on the constitutively active JMC-PTH1R mutants, both in vitro (43-45) and in vivo (46, 47). Moreover, our work highlights the osteocyte as a cellular target for therapeutic intervention, since these cells play a major role in calcium mobilization and the synthesis of key molecules including FGF23, DMP1, and sclerostin. The H223R-PTH1R mutation causes defects in osteocyte morphology and their canalicular system; whether normalizing gene expression in osteocytes can improve bone health in patients with JMC remains to be seen. It is conceivable that future clinical trials with JMC therapeutics may benefit from the evaluation of osteocyte morphology and function, which could provide additional endpoints for assessing the benefits of candidate drugs and their efficacy.

## Data Availability

Some or all datasets generated during and/or analyzed during the current study are not publicly available but are available from the corresponding author on reasonable request.

## Abbreviations

JMC: Jansen's metaphyseal chondrodysplasia
PTH: parathyroid hormone
PTHrP: PTH-related peptide
PTH1R: PTH/PTHrP receptor
